# Unravelling the Relationship Between Height, Lean Mass, Alzheimer’s Disease and Cognition Through Mendelian Randomization

**DOI:** 10.3390/genes16020113

**Published:** 2025-01-21

**Authors:** Jingxian Huang, Linxuan Zhang, Christopher Bodimeade, Malik Nassan, Dipender Gill, Héléne T. Cronjé, Marie-Joe Dib, Iyas Daghlas

**Affiliations:** 1 Department of Epidemiology and Biostatistics, School of Public Health, Imperial College London, London W12 0BZ, UK; jingxian.huang22@imperial.ac.uk; 2 Sequoia Genetics, Translation & Innovation Hub, London W12 0BZ, UK; linxuan.zhang@sequoiagenetics.com (L.Z.); christopher.bodimeade@sequoiagenetics.com (C.B.); 3 Mesulam Center for Cognitive Neurology and Alzheimer’s Disease, Northwestern University, Chicago, IL 60611, USA; malik.nassan@northwestern.edu; 4 MRC Biostatistics Unit, University of Cambridge, Cambridge CB2 0SR, UK; toinet.cronje@mrc-bsu.cam.ac.uk; 5 Cardiovascular Division, Perelman School of Medicine, University of Pennsylvania, Philadelphia, PA 19104, USA; marie-joe.dib@pennmedicine.upenn.edu; 6 UCSF Weill Institute for Neurosciences, Department of Neurology, University of California San Francisco, San Francisco, CA 94158, USA; iyas.daghlas@ucsf.edu

**Keywords:** cognition, Alzheimer’s disease, height, lean mass, Mendelian randomization

## Abstract

Background: Genetic evidence from Mendelian randomization (MR) analyses suggests that higher lean mass causally protects against Alzheimer’s disease (AD) and enhances cognitive function. However, the potential confounding role of height, which shares genetic etiology with lean mass, has not been fully examined. Methods: Genetic predictors of whole-body lean mass were obtained from a genome-wide association study (GWAS) performed in the UK Biobank cohort (UKB; *n* = 448,322). Genetic predictors of height were also obtained from UKB (height_0.5M_ = 455,332) and from a GWAS meta-analysis (height_1.5M_ *n* = 1,578,425). The study outcomes included clinically diagnosed AD (21,982 cases and 41,944 controls) and cognitive performance (*n* = 269,867). All study participants were of European ancestry. We conducted univariable and multivariable MR analyses to examine the total and independent effects of lean mass and height on the specified outcomes under different statistical adjustment strategies. Results: In univariable MR analyses, genetically proxied lean mass (odds ratio [OR] per 1-standard deviation [SD] increase 0.81, 95% confidence interval [CI] 0.72–0.91, *p* = 3.8 × 10^−4^) and height (OR 0.90, 95% CI 0.84–0.96, *p* = 0.001) were associated with reduced risk of AD. Genetically proxied lean mass (β 0.10, 95% CI 0.08–0.12, *p* = 6.24 × 10^−6^) and height (β 0.07, 95% CI 0.05–0.08, *p* = 1.16 × 10^−15^) were further associated with improved cognitive performance. In multivariable MR analyses, adjustment for height_1.5M_ partially attenuated the lean mass association with AD (OR 0.91, 95% CI 0.74–1.12, *p* = 0.40), whereas the height_1.5M_-AD association remained similar after adjusting for lean mass (OR 0.89, 95% CI 0.79–1.00, *p* = 0.04). Adjustment for height also attenuated the association of lean mass with cognitive performance (β 0.00, 95% CI −0.07–0.06, *p* = 0.94), whereas height maintained a similar association with improved cognitive performance after adjustment for lean mass (β 0.07, 95% CI 0.03–0.10, *p* = 0.001). Conclusions: Height may confound the genetic associations between lean mass and both cognitive performance and AD risk. Residual direct effects of lean mass on AD risk cannot be excluded due to limitations in statistical power and genetic instrument strength in MVMR. These findings emphasize the necessity of adjusting for height when using MR to investigate the clinical effects of lean mass.

## 1. Introduction

The identification of modifiable risk factors for Alzheimer’s disease (AD) is a key global health priority [1]. Sarcopenia, a state of low muscle mass, has been linked to poorer cognitive function and elevated dementia risk [2]. This association, documented primarily in cross-sectional observational studies, is consistent across clinical outcomes of minor cognitive impairment, AD dementia, and non-AD dementia [2]. In line with these epidemiological findings, our prior Mendelian randomization (MR) analysis found that genetically predicted lean mass, as a proxy for muscle mass, was associated with reduced risk of AD and with improved cognitive performance [3]. This analysis primarily utilized genetic variants associated with appendicular lean mass (ALM) adjusted for fat mass, with consistent findings for other lean mass measurements including whole-body lean mass [4].

Lean mass, which constitutes a major proportion of body tissue, typically increases with body size and accordingly has strong phenotypic [5] and genetic [4] correlations (r_g_ = 0.71) with height. Accounting for body size in epidemiologic studies of lean mass is thus important for estimating direct effects of lean mass on clinical outcomes [6]. Height is one such marker of body size and is preferred over others because it is relatively simple to measure and therefore affords large large-scale association data to be captured. We therefore previously incorporated multivariable MR analyses adjusting for genetic associations with height. This adjustment minimally impacted the MR findings, suggesting minimal confounding effects of height on the genetically proxied association of lean mass with AD. There are, however, several opportunities to extend this analysis. We previously used a smaller genome-wide association study (GWAS) [7] of height (*n* = 253,288) in order to minimize sample overlap with the lean mass GWAS, though larger GWASs have identified additional height-associated genetic variants that could enhance statistical power. Additionally, the use of a GWAS of ALM adjusted for fat mass may introduce collider bias; multivariable MR using unadjusted GWAS for lean mass and for height could avoid this bias [8]. Finally, our prior analysis focused on AD, and so we did not investigate the association of lean mass adjusted for height on cognitive performance. The present MR analysis therefore aims to systematically evaluate the potential confounding effect of height on the relationship between lean mass, AD risk, and cognitive performance.

## 2. Methods

### 2.1. Genetic Associations with Lean Mass and Height

Genetic associations with total lean mass (TLM), measured through bioelectrical impedance, were obtained from a GWAS performed in the UK Biobank (UKB) cohort [9]. This study included 448,322 participants of European ancestry, with a sample standard deviation (SD) of TLM of 11.5 kg. Genetic associations were adjusted for age, age^2^, sex, age × sex, age^2^ × sex, and the top 20 principal components of ancestry. Genetic associations are reported as SD changes in TLM per effect allele.

Height was used as a marker of body size because it is easy to measure and offers the largest genome-wide association study. We used genetic associations with height from two partially overlapping GWASs. The first was the largest GWAS meta-analysis of height from the Genetic Investigation of Anthropometric Traits (GIANT) consortium, which included 1,578,425 participants of European ancestry (height_1.5M_) [10]. Genetic associations, reported in SD units, were adjusted for age, sex, the top 20 principal components of ancestry, and study-specific covariates. The second GWAS included 455,332 participants of European ancestry from UKB (height_0.5M_) [9]. We selected this GWAS to enable multivariable MR analyses in a sample that was homogenous with the lean mass GWAS. Genetic associations are reported in SD units and were adjusted for age, age^2^, sex, age × sex, age^2^ × sex, and the top 20 principal components of ancestry.

We conducted secondary analyses examining height-adjusted lean mass GWAS in UKB [11]. These analyses included 297,908 unrelated UKB participants of White British ancestry. Participants were excluded if they met any of the following quality control criteria: sex chromosome aneuploidy, high heterozygosity rate, and absence of data on either height or TLM. Genetic association analyses were performed using linear regression in PLINK v2.0 [12]. The first GWAS directly modeled height as a covariate in the linear regression model (TLM_covariate_), while the second GWAS modeled TLM residualized on height (TLM_residual_). Effect sizes for both GWASs were standardized by dividing β estimates by the sample SD of TLM in UKB. Baseline study characteristics are reported in Supplementary Table S1.

### 2.2. Genetic Associations with Alzheimer’s Disease and Cognitive Performance

Summary statistics for clinically diagnosed AD were obtained from the Stage 1 GWAS meta-analysis from the International Genomics of Alzheimer’s Project (IGAP). The sample comprised 21,982 cases and 41,944 controls of European ancestry [13]. AD cases were diagnosed through clinical assessments, cognitive testing, or neuropathological examination. Genetic associations were adjusted for age, sex, and principal components of ancestry. For cognitive performance, we used summary statistics from a meta-analysis of 269,867 European ancestry individuals across 14 cohort studies [14]. A variety of neurocognitive tests were administered across the cohorts, spanning multiple domains that included memory, verbal reasoning, mathematical reasoning, and processing speed. These measures were standardized to derive a latent *g* factor, representing the shared variance among these tests and serving as a measure of general intelligence. Since the aim of this GWAS was to examine cognitive function in the general population, the studies did not use dementia screening tools such as the Mini-Mental State Examination. Genetic associations with cognitive performance were adjusted for age, sex, and principal components of ancestry, and are reported in SD units.

### 2.3. Selection of Genetic Proxies for Height and Lean Mass

For univariable MR analyses, we selected common (minor allele frequency > 0.01) single-nucleotide polymorphisms (SNPs) that were associated with the exposures at genome-wide statistical significance (*p*  <  5 × 10^−8^) and were available in the outcome datasets. Independent variants were identified through clumping using a 10 Mb window and linkage disequilibrium threshold r^2^ < 0.001, with reference to the 1000 Genomes European panel [15]. Genetic associations were harmonized to the same effect allele across exposures and outcomes. For palindromic variants, we used the default TwoSampleMR procedure to infer the forward strand when allele frequencies were unambiguous (minor allele frequency < 0.42). In multivariable MR analyses, we first pooled together genome-wide significant variants associated with either height or lean mass [16]. Given the disproportionate sample size of the height_1.5M_ GWAS relative to the TLM GWAS, we clumped this pooled list of variants using *p* values for associations with TLM.

### 2.4. Mendelian Randomization Analysis

We performed two-sample MR analyses to investigate the potential causal effects of genetically proxied lean mass and height on AD risk and cognitive performance. When investigating the total effect of the exposures in isolation, we used the random-effects inverse-variance weighted (IVW) method [15,17]. Valid causal inference from MR analyses depends on three assumptions: the genetic variants must be robustly associated with the exposure (relevance), have no common cause with the outcome (independence), and affect the outcome only through the exposure, and not through pleiotropic effects (exclusion restriction) [18]. To address the first assumption, we only selected variants associated with the exposures at genome-wide statistical significance. To address the second assumption, we ensured that all contributing GWASs included adjustment for principal components of ancestry, which minimizes the confounding of genetic associations by ancestry. To address violation of the exclusion restriction assumption, we conducted sensitivity analyses using MR models robust to various forms of pleiotropy, including weighted median estimation and MR-Egger regression [19,20]. As these statistical methods have varying degrees of statistical power, we primarily assessed for broad consistency of point estimates across the approaches.

We additionally used two statistical approaches to estimate the direct, adjusted effects of height and lean mass on the outcomes, accounting for mediating or pleiotropic genetic associations between the two exposures. Our main method was multivariable MR (MVMR), which was implemented using the regression-based MVMR approach in the TwoSampleMR R package [16]. We performed analyses separately adjusting genetically proxied lean mass for height_0.5M_ and for height_1.5M_. We report conditional F-statistics as measures of instrument strength in MVMR [21], with F-statistics less than 10 indicating potential weak instrument bias. In a secondary analysis, we performed conventional two-sample MR analyses using adjusted GWAS summary statistics. While intuitive, this approach could introduce collider bias that may affect the reliability of the MR estimates. Thus, the motivation for the inclusion of this analysis was to demonstrate the practical implications of different covariate adjustment approaches in MR.

Across analyses, statistical significance was defined as a two-tailed *p*-value less than 0.05.

### 2.5. Software

We used the TwoSampleMR v.0.5.7 and ieugwasr v.0.1.5 packages for all analyses [15]. Statistical codes used for this work are available from the corresponding author upon reasonable request.

## 3. Results

### 3.1. Univariable MR Estimates for Association of Height and Lean Mass with AD and Cognitive Performance

**Figure 1:**
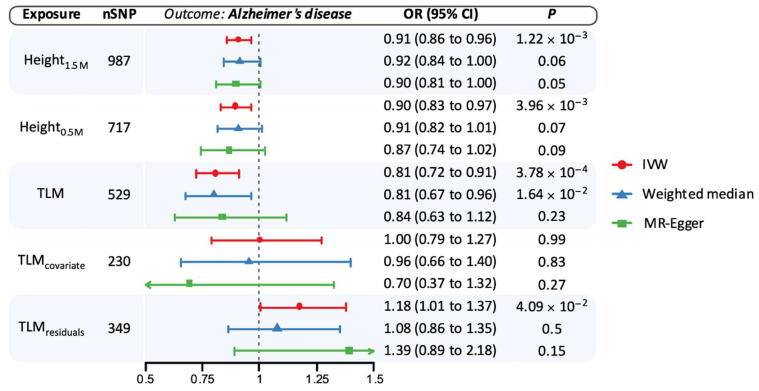
Univariable Mendelian randomization estimates for the association of genetically proxied height and lean mass with the risk of Alzheimer’s disease (AD). Estimates reflect the association of a standard deviation increase in genetically proxied levels of the exposure with risk of AD. Each shape corresponds to a different statistical model, with the position of the shape corresponding to the point estimate and bars corresponding to 95% confidence intervals. CI: Confidence interval; IVW: inverse-variance weighted; OR: odds ratio; SNP: single-nucleotide polymorphism; TLM: total body lean mass.

**Figure 2:**
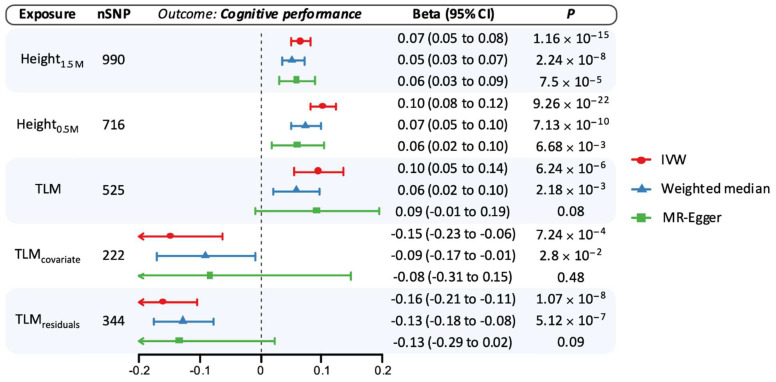
Univariable Mendelian randomization estimates for the association of genetically proxied height and lean mass with cognitive performance. Estimates reflect the association of a standard deviation (SD) increase in genetically proxied levels of the exposure with cognitive performance (SD units). Each shape corresponds to a different statistical model, with the position of the shape corresponding to the point estimate and bars corresponding to 95% confidence intervals. CI: Confidence interval; IVW: inverse-variance weighted; SNP: single-nucleotide polymorphism; TLM: total body lean mass.

Higher genetically proxied TLM was associated with a reduced risk of AD (odds ratio [OR] per 1-SD TLM 0.81, 95% confidence interval [CI] 0.72–0.91, *p* = 3.78 × 10^−4^) and with improved cognitive performance (β 0.10, 95% CI 0.08–0.12, *p* = 6.24 × 10^−6^; Figure 1 and Figure 2). These estimates were consistent when applying the weighted median and MR Egger methods (Figure 1 and Figure 2). Genetically proxied height_1.5M_ was also associated with a reduced risk of AD (OR per 1-SD taller height 0.91, 95% CI 0.86–0.96, *p* = 1.22 × 10^−3^) and with improved cognitive performance (β 0.07, 95% CI 0.05–0.08, *p* = 1.16 × 10^−15^; Figure 1 and Figure 2). Findings were similar in sensitivity analyses (Figure 1 and Figure 2). Scatter plots of genetic associations between variants, exposures, and outcomes are shown in Supplementary Figures S1–S10.

### 3.2. Estimating the Direct Effect of Lean Mass and Height on AD and Cognitive Performance

**Figure 3:**
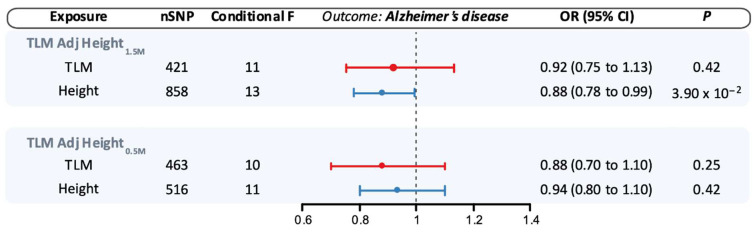
Multivariable Mendelian randomization estimates for the association of genetically proxied height and lean mass with the risk of Alzheimer’s disease (AD). Estimates reflect the association of a standard deviation increase in genetically proxied levels of the exposure with risk of AD. Shapes reflect point estimates, and bars correspond to 95% confidence intervals. CI: Confidence interval; OR: odds ratio; SNP: single-nucleotide polymorphism; TLM: total body lean mass.

When adjusting for genetically proxied height_1.5M_ in MVMR analyses, the association between genetically proxied TLM and AD was attenuated compared to the univariable estimate (OR 0.92, 95% CI 0.75–1.13, *p* = 0.42), with similar findings when adjusting for genetically proxied height_0.5M_ (Figure 3). In contrast, adjustment for genetically proxied lean mass did not substantively modify the point estimate for the association between genetically proxied height_1.5M_ and AD (OR 0.88, 95% CI 0.78–0.99, *p* = 0.04), with similar findings for genetically proxied height_0.5M_ (Figure 3).

**Figure 4:**
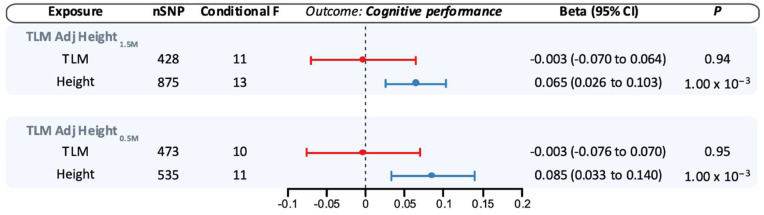
Multivariable Mendelian randomization estimates for the association of genetically proxied height and lean mass with cognitive performance. Estimates reflect the association of a standard deviation (SD) increase in genetically proxied levels of the exposure with cognitive performance (SD units). Shapes reflect point estimates, and bars correspond to 95% confidence intervals. CI: Confidence interval; SNP: single-nucleotide polymorphism; TLM: total body lean mass.

When adjusting for genetically proxied height_1.5M_ in MVMR analyses, the association between genetically proxied TLM and cognitive performance was attenuated towards the null (β −0.003, 95% CI −0.07–0.06, *p* = 0.94), with similar findings when adjusting for genetically proxied height_0.5M_ (Figure 4). As with AD, adjustment for genetically proxied lean mass did not significantly alter the point estimate for the association of genetically proxied height_1.5M_ with cognitive performance (β 0.065, 95% CI 0.03–0.10, *p* = 0.001), and findings were similar when adjusting for genetically proxied height_0.5M_ (Figure 4). Despite large numbers of proxy variants for the exposures, the conditional F-statistics for height (13) and lean mass (11) were relatively low in these analyses (Figure 3 and Figure 4).

We conducted secondary MR analyses using a GWAS for lean mass adjusted for height (Figure 1 and Figure 2). Lean mass adjusted for height had a null MR estimate with AD, though confidence intervals were relatively wide compared to findings from the unadjusted analyses (OR 1.00, 95% CI 0.79–1.27, *p* = 0.99). In contrast, MR estimates using the GWAS of residuals after regressing lean mass on height (i.e., TLM_residuals_) supported an increased risk of AD (OR 1.18, 95% CI 1.01–1.37, *p* = 0.04). MR analyses of genetically proxied height-adjusted lean mass also demonstrated adverse associations with cognitive performance, regardless of the adjustment method (Figure 2).

## 4. Discussion

In this MR analysis, we identified associations of higher genetically proxied lean mass and height with the reduced risk of clinically diagnosed AD and with improved cognitive performance. In MVMR analyses, the association between lean mass and AD was partially attenuated upon adjusting for height, whereas the relationship between lean mass and cognition was fully attenuated. By contrast, the associations between genetically proxied lean mass and both AD risk and cognitive performance showed opposite directionality when using height-adjusted genome-wide association studies. These results yield several key insights.

First, findings from MVMR analyses suggest that height (as a measure of total body size) partially confounds the association between lean mass and AD. The lean mass–AD MR estimate diminished by approximately 50% on the log-odds scale after adjusting for genetically proxied height, with consistent results from analyses leveraging different height GWAS data. However, conditional F-statistics approached the weak instrument threshold of 10, suggesting that collinearity between height and lean mass may have reduced statistical power and introduced bias up to 10% of the confounded observational estimate [21]. The resulting confidence intervals encompassed the univariable MR estimates, precluding definitive conclusions about differences between the adjusted and unadjusted analyses. Thus, while these findings weaken the strength of evidence for the association of lean mass with AD, they do not exclude a residual causal effect. In contrast, adjustment for height in MVMR analyses completely nullified the association between lean mass and cognitive performance. Although subject to similar potential biases, this degree of attenuation provides stronger statistical evidence that height confounds the lean mass–cognitive performance relationship. Taken together, these findings underscore the importance of accounting for total body size in epidemiologic investigations of lean mass.

The height-adjusted GWAS analyses showed opposite directions of associations for genetically proxied lean mass with AD and cognitive performance compared to MVMR results. This finding is difficult to reconcile mechanistically and conflicts with the protective effects observed for both height and lean mass in univariable Mendelian randomization analyses, suggesting potential limitations of this adjustment approach. Indeed, prior work has demonstrated that covariate adjustment in GWASs can introduce collider bias [8]. Such bias can be avoided through multivariable MR analyses [8], which underscores the importance of carefully triangulating findings across different methodological approaches. We also note that, due to data availability, the covariate-adjusted GWAS used a smaller UKB sample compared to the unadjusted analyses. Discrepant findings across the analytic methods may thus also stem from differences in identified genetic instruments.

These findings support a potential causal effect of height on reduced risk of AD and improved cognitive performance. This constitutes a replication of a prior MR analysis that leveraged the height_0.25M_ sample and a smaller AD GWAS of 17,008 cases [7,13,22]. This prior study estimated an OR of 0.89, closely aligning with the OR of 0.91 estimated in our analysis. We extend this finding by demonstrating that this association is not biased by pleiotropic effects of genetic proxies on lean mass. The association of height with improved cognitive performance has also been reported in prior genetic analyses [23] and in meta-analyses of prospective cohort studies [24]. One proposed mechanism for these associations is that height reflects upregulated growth and developmental processes, potentially mediated through IGF-1 and growth hormone signaling, which in turn may influence cognition [24,25]. These signaling pathways may also influence the growth and development of cortical structures that are relevant to AD pathogenesis. This hypothesis is supported by prior epidemiologic investigations which have shown associations of height with regional grey matter volume in the temporal lobes and prefrontal cortices [25]. Finally, height may also influence AD risk through its effects on educational attainment and socioeconomic status, which have been hypothesized to increase cognitive reserve [26,27]. Although our study was not designed to identify the specific biological pathways linking height to Alzheimer’s disease risk, future multiomics investigations could test these hypotheses.

There are several strengths to highlight from the present analysis. The MR approach, relative to conventional epidemiologic analyses, mitigates bias due to residual confounding and reverse causality. We evaluated multiple approaches for adjusting genetic associations with height, reducing the likelihood that our findings stem from characteristics unique to any single height GWAS or statistical approach. We also restricted analysis to clinically diagnosed AD, which avoids bias induced by inclusion of AD ‘proxy’ cases in larger GWASs [28]. Moreover, the AD GWAS does not include any participants who participated in UKB, mitigating the risk of bias arising from sample overlap in MR analyses.

There are also limitations to consider. First, prior studies have shown that associations of height with cognitive phenotypes, including educational attainment, are markedly attenuated when using within-family designs that minimize assortative mating bias [29]. As our summary-level analysis cannot account for assortative mating, this relationship requires further investigation using study designs that can address this potential bias. Similarly, despite consistent findings in sensitivity analyses, we cannot exclude bias due to pleiotropy or to confounding of the genetic associations with the outcomes. As height GWAS sample sizes increase, newly discovered variants with smaller effects may influence height indirectly through upstream traits that confound the height–AD relationship; including such variants in MVMR may introduce bias. Second, these findings were conducted using data from European populations and may therefore have limited generalizability to other ancestry groups. Third, the impedance-based measurement of lean mass serves as an indirect measure of muscle mass, potentially introducing bias if the relationship between impedance and muscle mass varies systematically across population subgroups. Fourth, in contrast to conventional observational analyses, the summary-level Mendelian randomization design cannot assess non-linear relationships between height or lean mass and outcomes. Fifth, these associations reflect the effects of lifelong differences in lean mass rather than changes occurring during specific periods of life. Sixth, the height_1.5M_ GWAS had relatively poor genomic coverage, which restricted the number of genetic variants available for use in MR. Finally, the interpretation of findings from MVMR analyses was limited by statistical power (resulting in relatively wider confidence intervals) and by conditional instrument strength. These limitations may have obscured potential direct effects of lean mass on AD and cognitive performance. Future methodological research should develop approaches to address these challenges, particularly when exposures have unequal numbers of genetic instruments in MVMR.

## 5. Conclusions

These findings suggest that height confounds the genetic associations between lean mass and both cognitive performance and AD risk. These findings emphasize the necessity of adjusting for height when using MR to investigate the clinical effects of lean mass. Future investigations should focus on addressing potential biases in multivariable MR related to conditional instrument strength and assortative mating.

## Data Availability

Data used for this analysis are publicly available.

## Supplementary Materials

The following supporting information can be downloaded at https://www.mdpi.com/article/10.3390/genes16020113/s1, Table S1: Baseline characteristics of UK Biobank participants included in adjusted genome-wide association studies for total lean mass (N=297,908), Figure S1: Scatter plot for univariable MR analysis of total lean mass (TLM) on Alzheimer’s disease (AD), Figure S2: Scatter plot for univariable MR analysis of total lean mass (TLM) on cognitive performance (CP), Figure S3: Scatter plot for univariable MR analysis of height_1.5M_ on Alzheimer’s disease (AD), Figure S4: Scatter plot for univariable MR analysis of height_1.5M_ on cognitive performance (CP), Figure S5: Scatter plot for univariable MR analysis of height_0.5M_ on Alzheimer’s disease (AD), Figure S6: Scatter plot for univariable MR analysis of height_0.5M_ on cognitive performance (CP), Figure S7: Scatter plot for univariable MR analysis of total lean mass (TLM_covariate_) on Alzheimer’s disease (AD), Figure S8: Scatter plot for univariable MR analysis of total lean mass (TLM_covariate_) on cognitive performance (CP), Figure S9: Scatter plot for univariable MR analysis of total lean mass (TLM_residual_) on Alzheimer’s disease (AD), Figure S10: Scatter plot for univariable MR analysis of total lean mass (TLM_residual_) on cognitive performance (CP).
