# Supplementary material for: Unravelling the Relationship Between Height, Lean Mass, Alzheimer’s Disease and Cognition Through Mendelian Randomization
Source: Genes (Basel). 2025 Jan 21;16(2):113. doi: 10.3390/genes16020113 (PMC11855215; doi:10.3390/genes16020113)

Table of Contents

Supplementary Table 1. Baseline characteristics of UK Biobank participants included in adjusted genome-wide association studies for total lean mass (N=297,908).

Supplementary Figure 1. Scatter plot for univariable MR analysis of total lean mass (TLM) on Alzheimer’s disease (AD).

Supplementary Figure 2. Scatter plot for univariable MR analysis of total lean mass (TLM) on cognitive performance (CP).

Supplementary Figure 3. Scatter plot for univariable MR analysis of $height_{1.5M}$ on Alzheimer’s disease (AD).

Supplementary Figure 4. Scatter plot for univariable MR analysis of $height_{1.5M}$ on cognitive performance (CP).

Supplementary Figure 5. Scatter plot for univariable MR analysis of $height_{0.5M}$ on Alzheimer’s disease (AD).

Supplementary Figure 6. Scatter plot for univariable MR analysis of $height_{0.5M}$ on cognitive performance (CP).

Supplementary Figure 7. Scatter plot for univariable MR analysis of total lean mass ($TLM_{covariate}$) on Alzheimer’s disease (AD).

Supplementary Figure 8. Scatter plot for univariable MR analysis of total lean mass ($TLM_{covariate}$) on cognitive performance (CP).

Supplementary Figure 9. Scatter plot for univariable MR analysis of total lean mass ($TLM_{residual}$) on Alzheimer’s disease (AD).

Supplementary Figure 10. Scatter plot for univariable MR analysis of total lean mass ($TLM_{residual}$) on cognitive performance (CP).

Supplementary Table 1. Baseline characteristics of UK Biobank participants included in adjusted genome-wide association studies for total lean mass (N=297,908). Continuous variables are summarized as mean ± standard deviation or median (interquartile range). Categorical variables are summarized as N (%).

	**Participants (N=297,908)**
**Age at blood draw, y**	56.74 ± 7.97
**Female, N**	160,077 (53.73%)
**Body mass index, kg/m^2**	27.36 ± 4.75
**Height, cm**	168.89 ± 9.23
**Appendicular lean mass, kg**	23.78 ± 5.64
**Total lean mass, kg**	53.52 ± 11.53
**Blood pressure, mmHg**	
Systolic blood pressure	139.99 ± 19.66
Diastolic blood pressure	82.21 ± 10.66
**Smoking status, N**	
Never	162,575 (54.57%)
Previous	104,274 (35.00%)
Current	30,066 (10.09%)
**Diabetes Mellitus**	22,563 (7.57%)
**Medication use, N**	
Cholesterol-lowering medication	32,733 (10.98%)
Antihypertensive medication	30,562 (10.25%)
**Blood biochemistry**	
Glycated haemoglobin (HbA1c, %)	35.90 (6.40)
Total cholesterol (mmol/L)	4.58 ± 0.93
LDL cholesterol (mmol/L)	1.71 ± 0.43
HDL cholesterol (mmol/L)	1.30 ± 0.32
Triglycerides (mmol/L)	1.48 (1.05 - 2.15)
Creatinine (umol/L)	0.06 ± 0.01

Supplementary Figure 1. Scatter plot for univariable MR analysis of total lean mass (TLM) on Alzheimer's disease (AD). Y-axis represents effects of genetic variants on AD; X-axis represents effects of genetic variants on TLM. SNP: single nucleotide polymorphism.

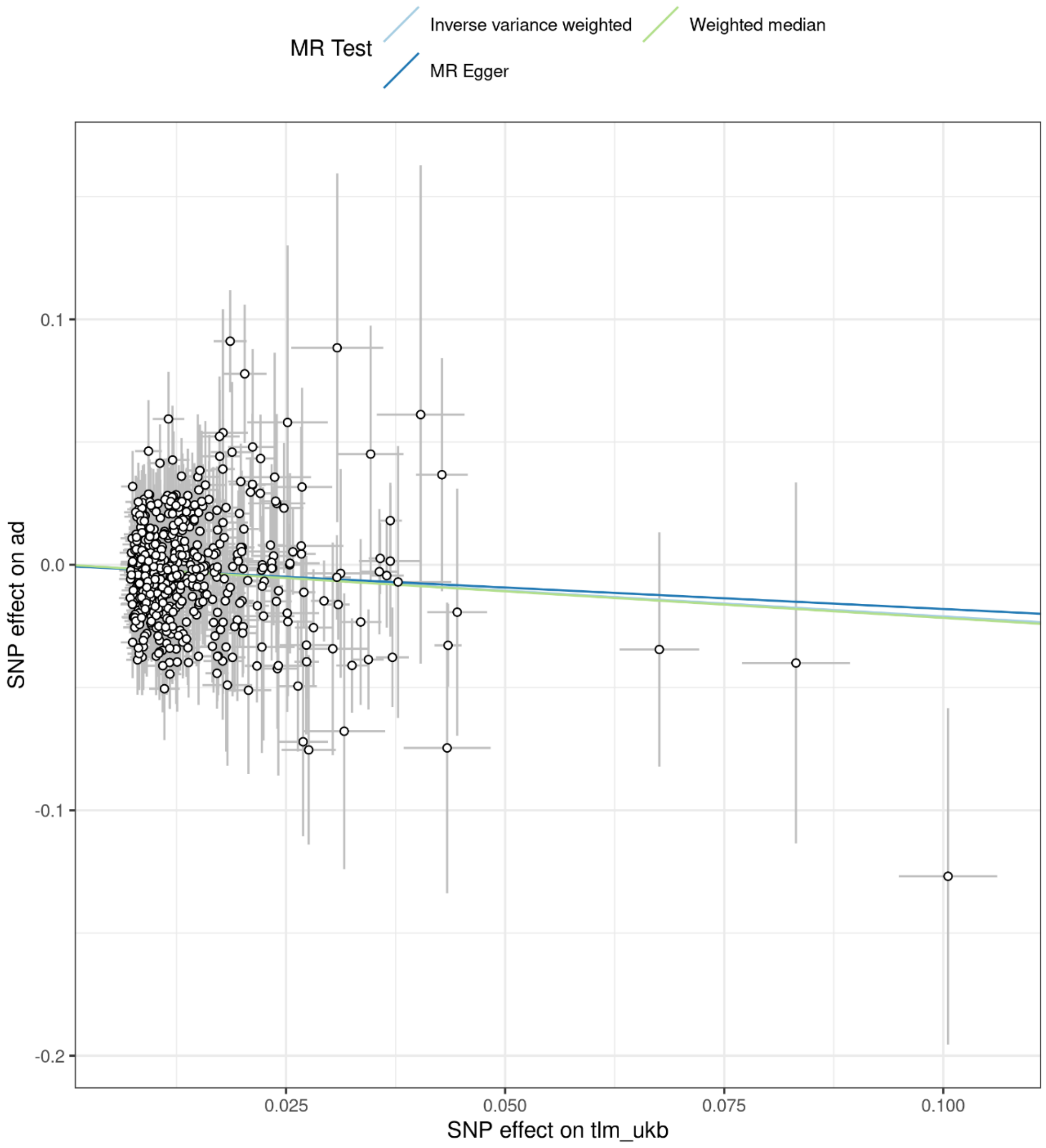

Supplementary Figure 2. Scatter plot for univariable MR analysis of total lean mass (TLM) on cognitive performance (CP). Y-axis represents effects of genetic variants on CP; X-axis represents effects of genetic variants on TLM. SNP: single nucleotide polymorphism.

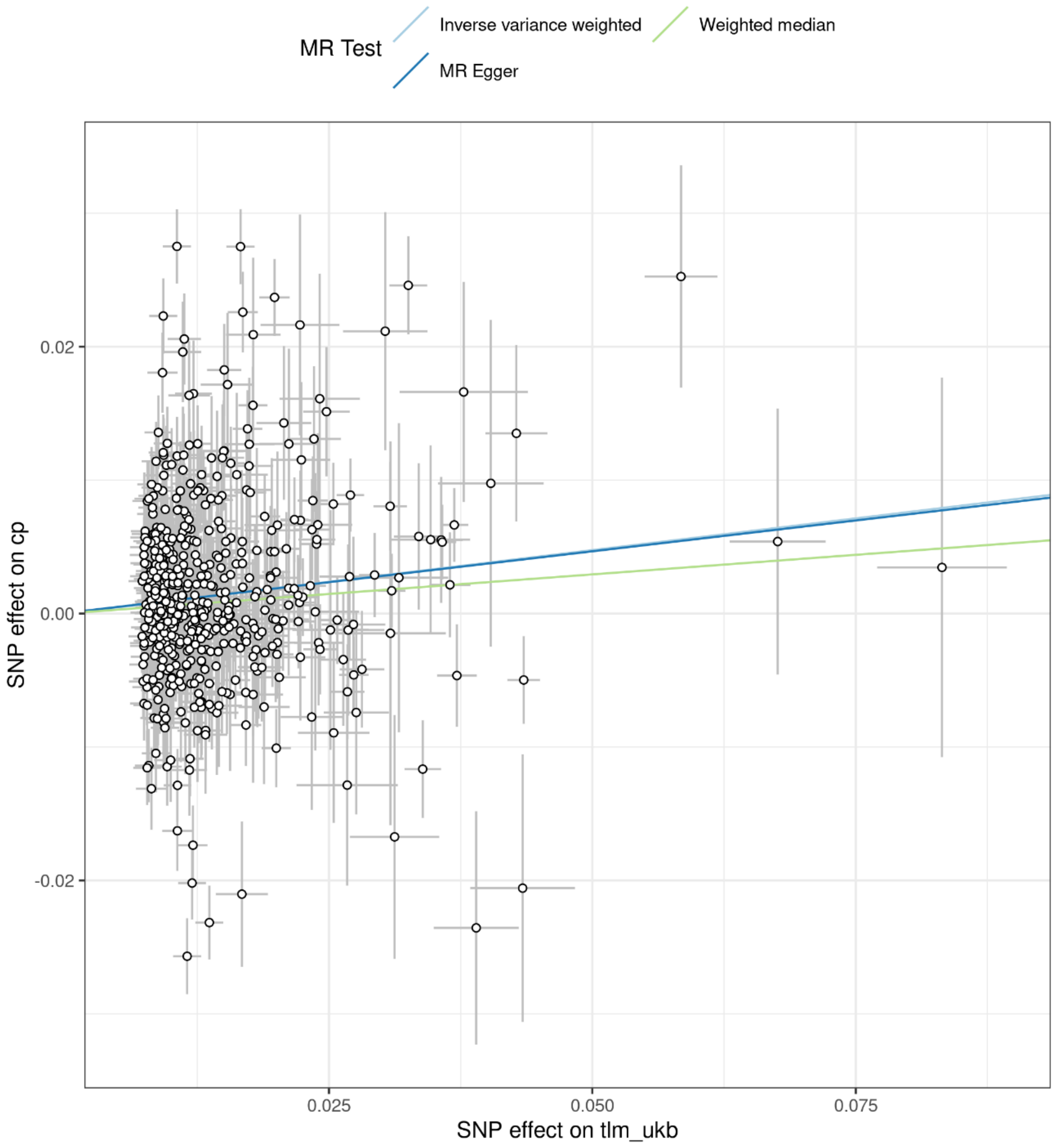

Supplementary Figure 3. Scatter plot for univariable MR analysis of height$_{1.5M}$ on Alzheimer's disease (AD). Y-axis represents effects of genetic variants on AD; X-axis represents effects of genetic variants on height$_{1.5M}$. SNP: single nucleotide polymorphism.

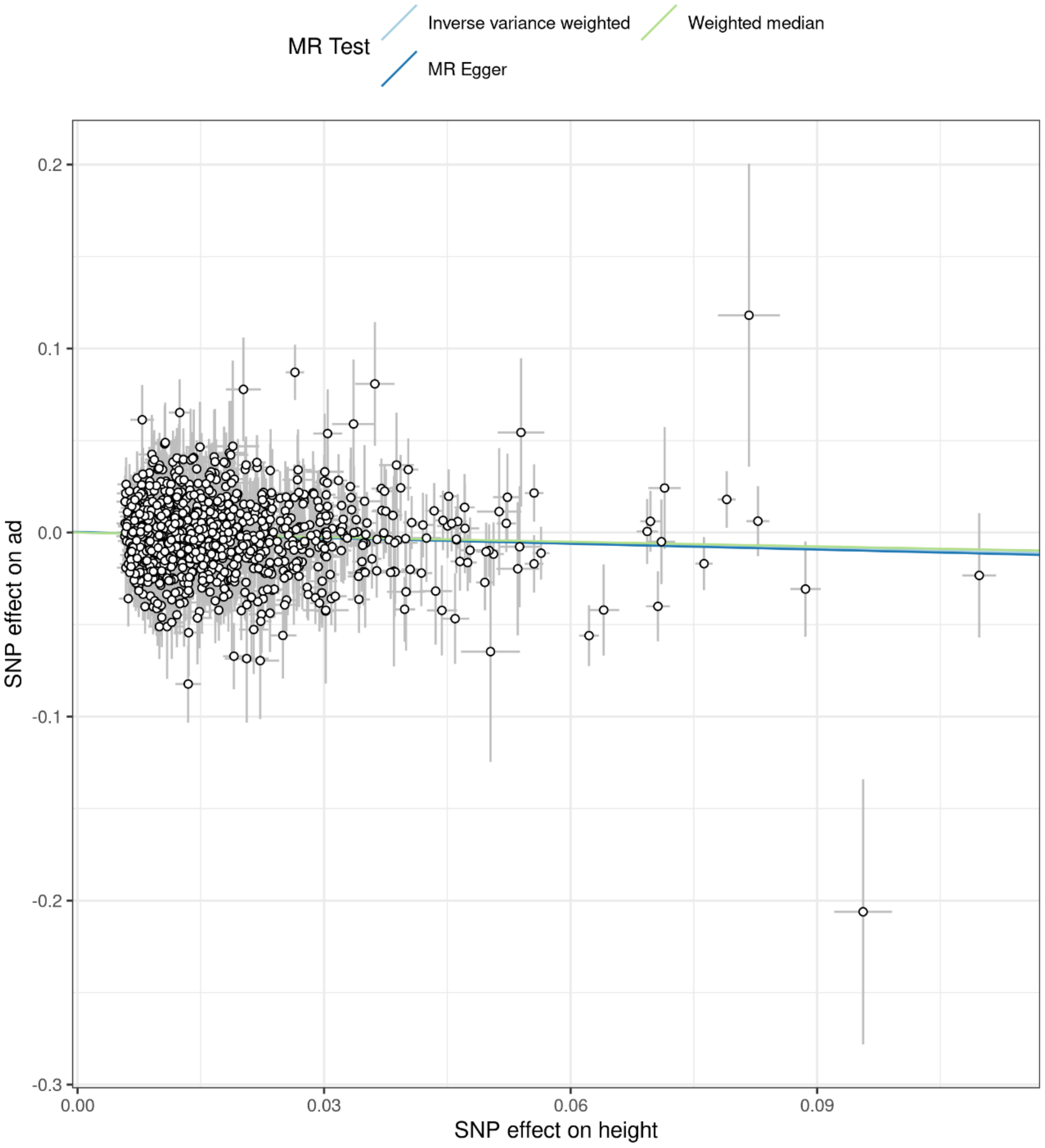

Supplementary Figure 4. Scatter plot for univariable MR analysis of height$_{1.5M}$ on cognitive performance (CP). Y-axis represents effects of genetic variants on CP; X-axis represents effects of genetic variants on height$_{1.5M}$. SNP: single nucleotide polymorphism.

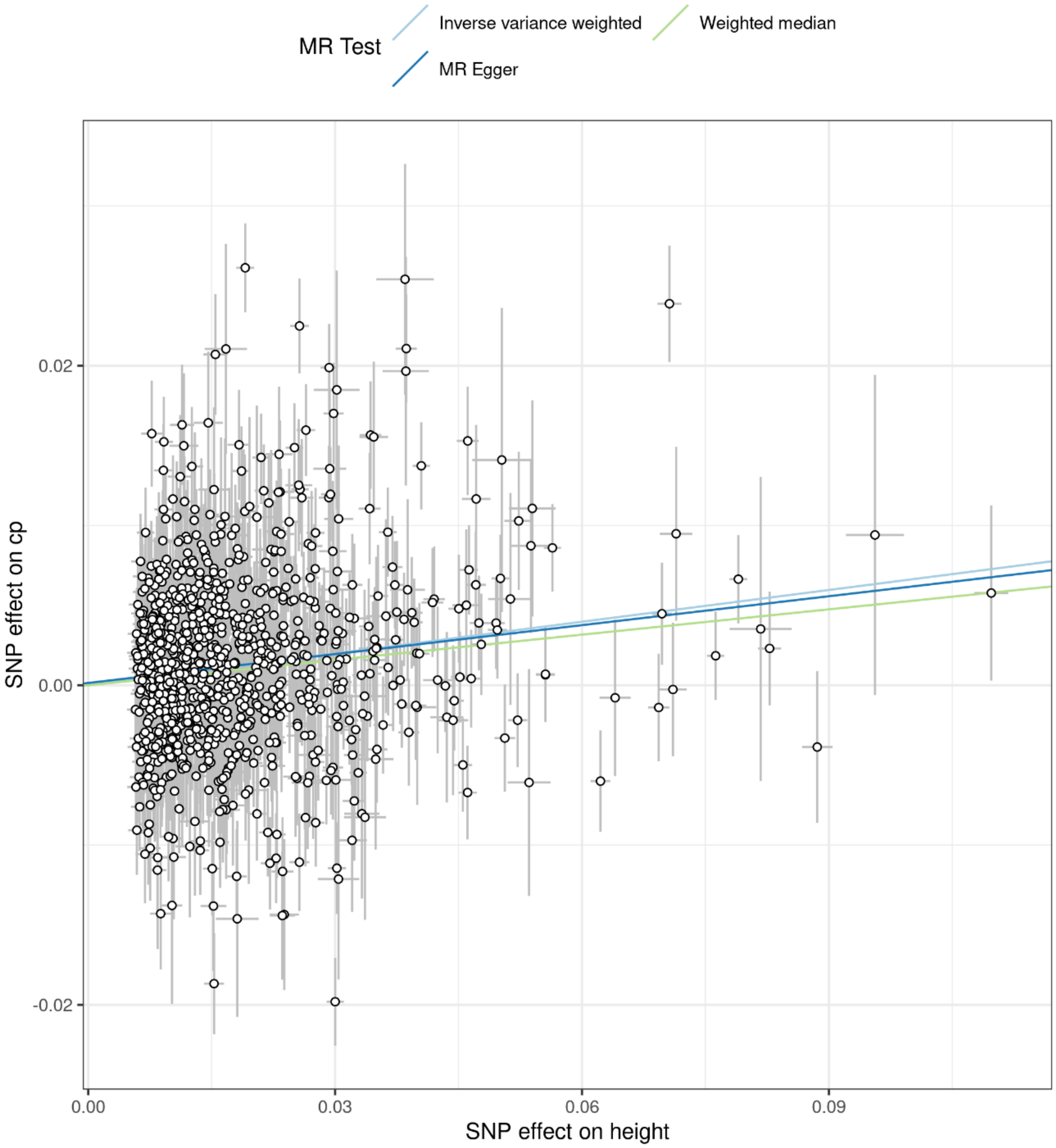

Supplementary Figure 5. Scatter plot for univariable MR analysis of height$_{0.5M}$ on Alzheimer's disease (AD). Y-axis represents effects of genetic variants on AD; X-axis represents effects of genetic variants on height$_{0.5M}$. SNP: single nucleotide polymorphism.

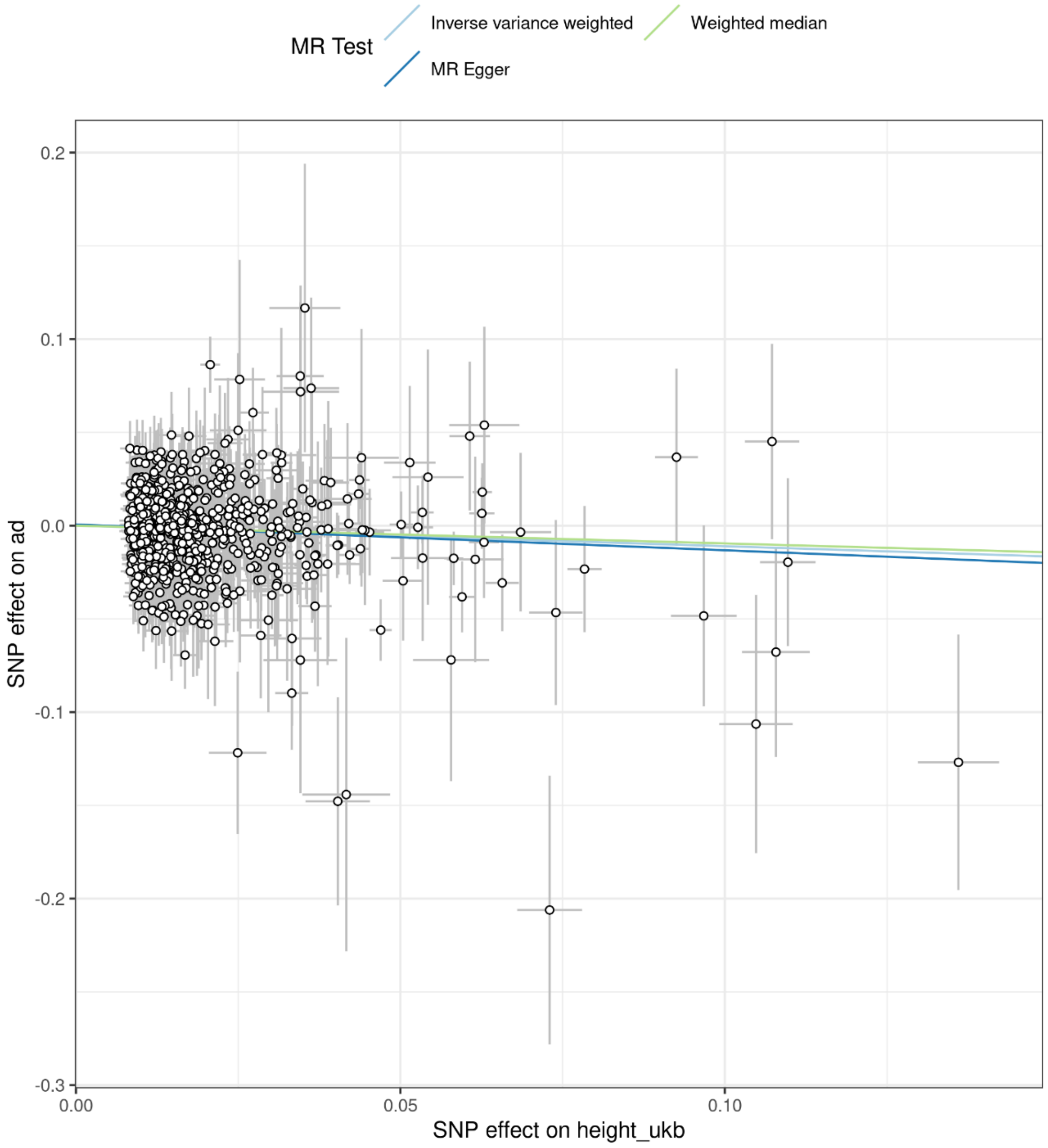

Supplementary Figure 6. Scatter plot for univariable MR analysis of height$_{0.5M}$ on cognitive performance (CP). Y-axis represents effects of genetic variants on CP; X-axis represents effects of genetic variants on height$_{0.5M}$. SNP: single nucleotide polymorphism.

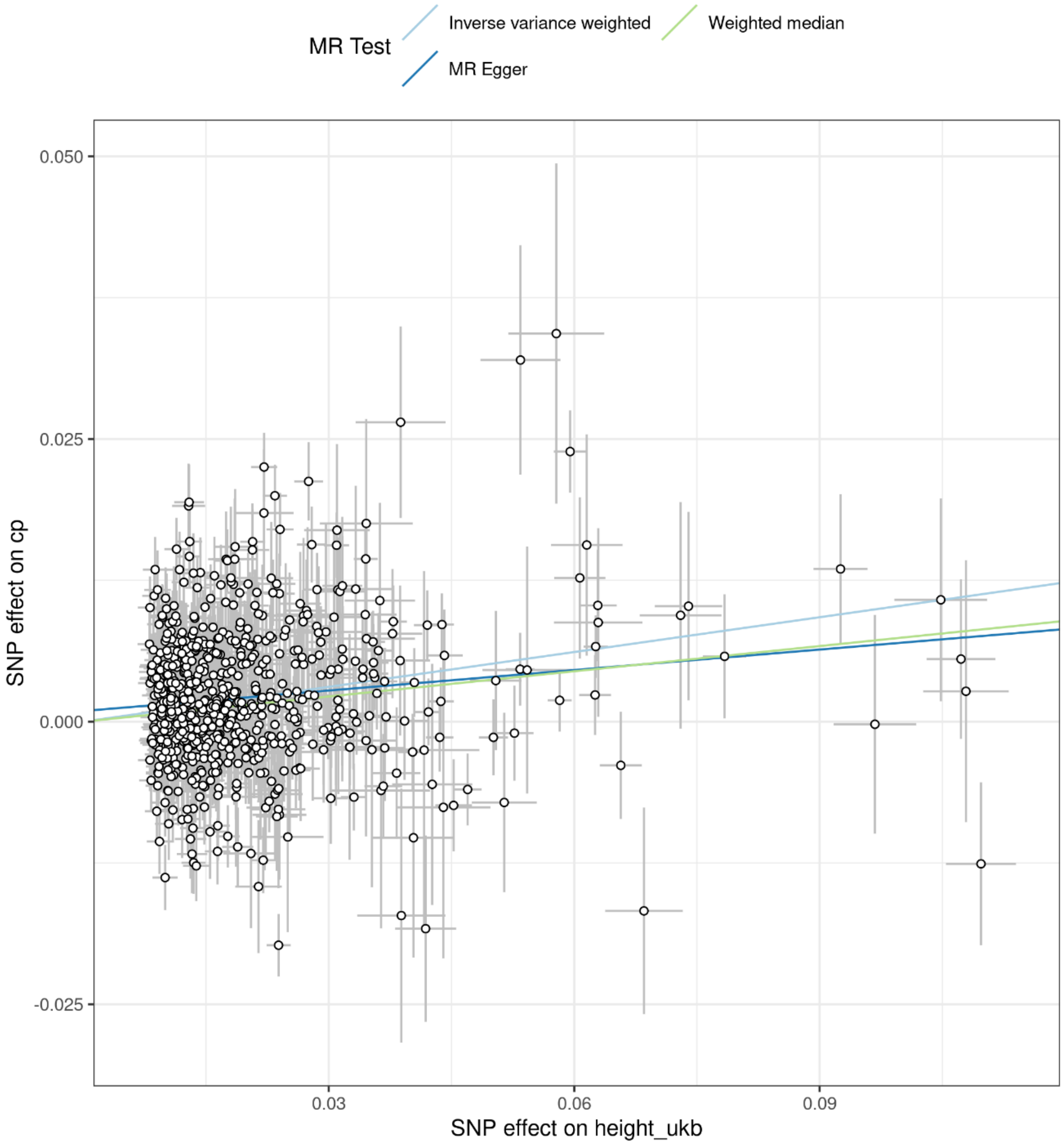

Supplementary Figure 7. Scatter plot for univariable MR analysis of total lean mass ($TLM_{covariate}$) on Alzheimer's disease (AD). Y-axis represents effects of genetic variants on AD; X-axis represents effects of genetic variants on $TLM_{covariate}$. SNP: single nucleotide polymorphism.

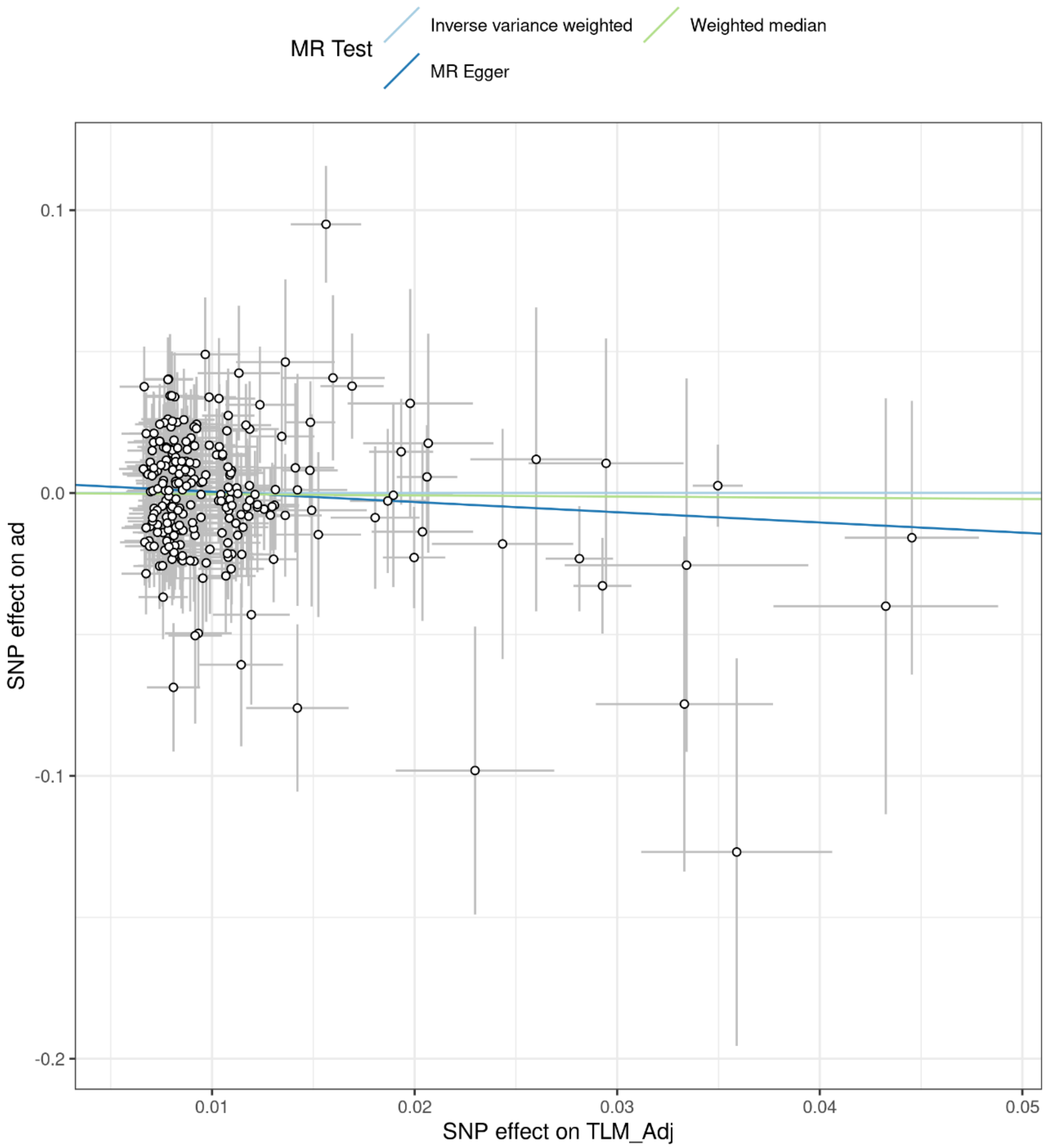

Supplementary Figure 8. Scatter plot for univariable MR analysis of total lean mass ($TLM_{covariate}$) on cognitive performance (CP). Y-axis represents effects of genetic variants on CP; X-axis represents effects of genetic variants on $TLM_{covariate}$. SNP: single nucleotide polymorphism.

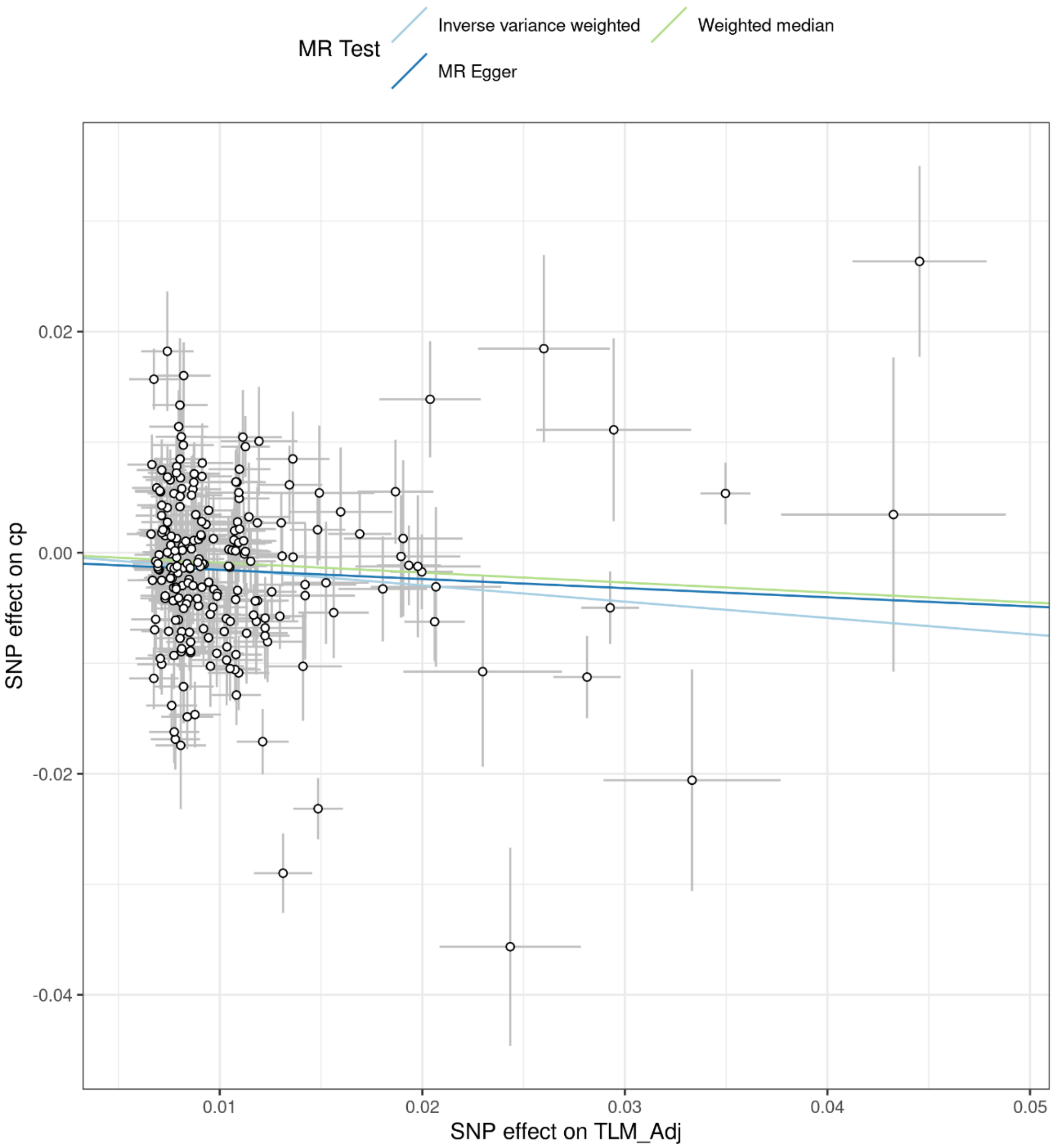

Supplementary Figure 9. Scatter plot for univariable MR analysis of total lean mass ($TLM_{residual}$) on Alzheimer's disease (AD). Y-axis represents effects of genetic variants on AD; X-axis represents effects of genetic variants on $TLM_{residual}$. SNP: single nucleotide polymorphism.

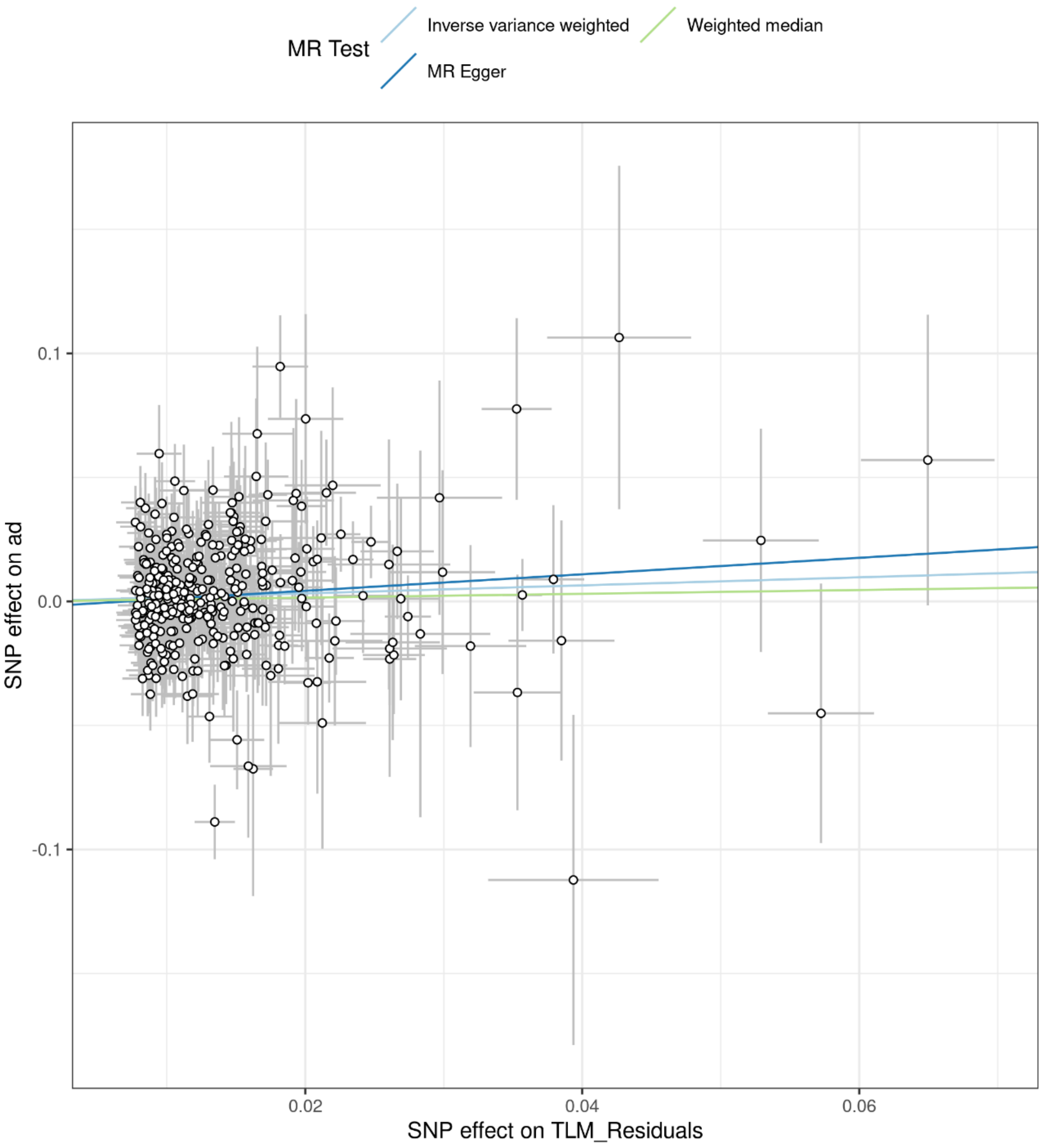

Supplementary Figure 10. Scatter plot for univariable MR analysis of total lean mass ($TLM_{residual}$) on cognitive performance (CP). Y-axis represents effects of genetic variants on CP; X-axis represents effects of genetic variants on $TLM_{residual}$. SNP: single nucleotide polymorphism.

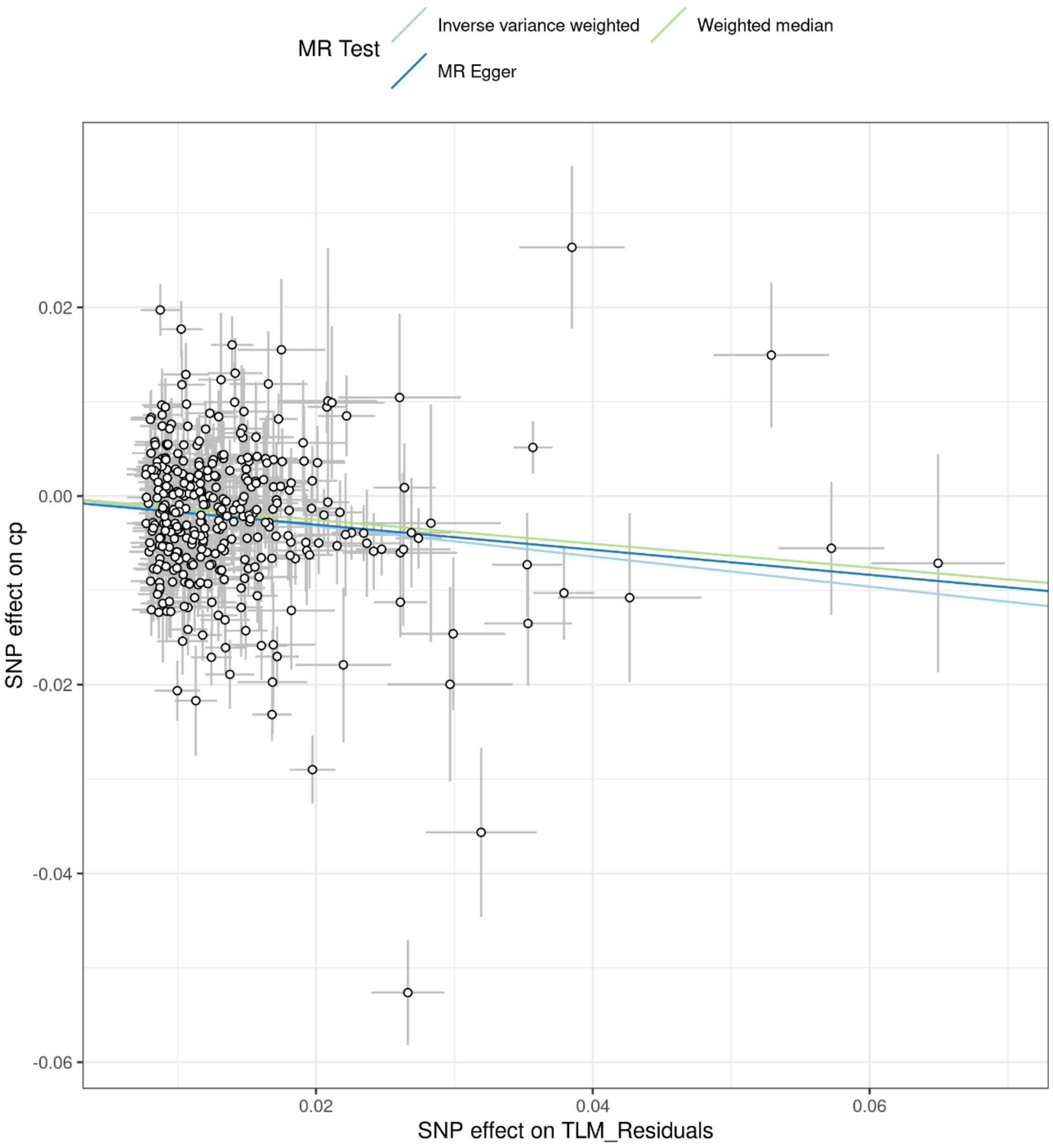

Supplement: Supplementary file 1 [file genes-16-00113-s001.zip › genes-3371743-supplementary.pdf]
